# The Psychosocial and Healthcare Utilisation Experiences of Women With Gestational Diabetes Mellitus From Diagnosis to 12 Months Postnatal: A Protocol for a Qualitative, Longitudinal Interview Study

**DOI:** 10.1002/mpr.70038

**Published:** 2025-09-11

**Authors:** Madeleine Benton, Elana Payne, Nabiha Waheed, Katrina Turner, Helen R. Murphy, Helen Skouteris, Khalida Ismail, Sergio A. Silverio

**Affiliations:** ^1^ Department of Psychological Medicine Institute of Psychiatry Psychology and Neuroscience King's College London London UK; ^2^ Department of Women & Children's Health School of Life Course & Population Sciences London UK; ^3^ Faculty of Life Sciences & Medicine King's College London London UK; ^4^ Department of Psychology Institute of Population Health Liverpool UK; ^5^ Faculty of Health and Life Sciences University of Liverpool Liverpool UK; ^6^ Population Health Sciences Bristol Medical School University of Bristol Bristol UK; ^7^ Norwich Medical School University of East Anglia Norwich UK; ^8^ School of Public Health and Preventive Medicine Monash University Melbourne Australia; ^9^ Warwick Business School University of Warwick Coventry UK

**Keywords:** gestational diabetes mellitus, perinatal health, pregnancy, qualitative longitudinal

## Abstract

**Objectives:**

Gestational diabetes mellitus (GDM) affects a significant and growing proportion of women each year. We know little about how their needs and experiences change over the perinatal period. This study aims to explore the psychosocial and healthcare experiences of women with a GDM diagnosis from pregnancy to 12‐month postnatal.

**Methods:**

This is a qualitative, longitudinal study comprising of individual interviews with women diagnosed with GDM who live in the UK at three time points: 35‐weeks’ gestation; 3‐months postnatal; 12‐months postnatal. Data will be analysed using template analysis. Comparisons will be made both between participants and timepoints to allow for biographical and temporal interpretations.

**Results:**

This study will provide in‐depth insights into women's lived‐experiences, psychosocial needs, and interactions with healthcare services. Particular attention will be given to the transition between antenatal and postnatal periods.

**Conclusion:**

Study findings will support the co‐development of a tailored mother‐infant dyad intervention grounded in women's needs and preferences across the perinatal period, aimed at improving long‐term health outcomes for women and their children with a focus on diabetes prevention.

## 1 Introduction

Gestational diabetes mellitus (GDM) is characterised by hyperglycaemia, which is first recognised during pregnancy (Hod et al. 2015). GDM is one of the most common pregnancy complications, affecting approximately 14% of pregnancies globally, with rates continuing to rise (Wang et al. 2022). In the short‐term, GDM is associated with adverse obstetric, maternal and neonatal outcomes, such as pre‐eclampsia, caesarean section, pre‐term delivery, foetal macrosomia (excessive birthweight), and neonatal intensive care unit admission (Ye et al. 2022). The potential short‐term risks of GDM can be significantly reduced or managed with health behaviour change (including to diet and physical activity), alongside monitoring of blood glucose levels, attendance at additional medical appointments for close monitoring and early detection of more severe risks, and for some, the addition of pharmacological management using insulin and/or metformin (American Diabetes Association 2021; National Institute of Health Care Excellence 2015). Consequently, GDM pregnancies can become highly medicalised (Diane et al. 2017).

Women who have been diagnosed with GDM have a 50% increased risk for GDM recurrence in subsequent pregnancies and have an up to 10‐fold increased risk of type 2 diabetes mellitus (T2DM) compared to women without GDM (Bellamy et al. 2009; Vounzoulaki et al. 2020). Long‐term foetal outcomes of GDM, include increased rates of obesity and T2DM in both childhood and adulthood (Murray and Reynolds 2020). The health, social, and economic consequences of T2DM are severe and it is associated with a reduction in life expectancy of 10 years (Kaptoge et al. 2023). Interventions targeting behaviour change (e.g. diet and physical activity) in the postnatal period can reduce the risk of T2DM for women (N. Li et al. 2021). Such interventions are widely known as diabetes prevention programmes (DPPs). A diagnosis of GDM signals long‐term metabolic risk and presents a unique opportunity for early intervention. Once considered a condition of late pregnancy, evidence now shows that GDM has origins before conception and lasting effects beyond birth, prompting calls for both research and care to adopt a lifecourse approach rather than focusing solely on pregnancy (Buchanan et al. 2025; Hivert et al. 2024; Simmons et al. 2024).

Evidence from randomised controlled trials (RCTs) suggests intensive DPPs can reduce the risk of T2DM in the general population by 58% (G. Li et al. 2008; Lindström et al. 2006; Tuomilehto et al. 2001). In the GDM population, a meta‐analysis of DPPs observed a 24% reduction in the incidence of T2DM compared with standard care (Retnakaran et al. 2023). However, DPPs are often difficult to implement as they are resource and cost intensive, tested in highly controlled settings, therefore limiting their real‐world applicability (Dasgupta et al. 2018).

Qualitative studies have identified barriers to DPP uptake for women in the perinatal period including competing demands of motherhood, early resumption of work, limited family support and a tendency to prioritise infant care over maternal care (Dennison et al. 2019; Van Ryswyk et al. 2015). These are further compounded by the absence of structured postpartum follow‐up in many healthcare systems, contributing to low rates of postpartum glucose testing (Dunne et al. 2024).

Many DPPs for women after GDM have primarily targeted diet and physical activity, often overlooking behaviours and barriers specific to the postnatal period. Breastfeeding for example, can reduce the risk of T2DM in women with prior GDM by over 20% (Aune et al. 2014; Chowdhury et al. 2015; Ley et al. 2020). Yet, it remains under‐addressed in DPP design, despite its well‐established benefits for both maternal and infant health (Horta et al. 2015; Nguyen et al. 2019; Tarrant et al. 2020; Victora et al. 2016). Further, despite the recognised impact of GDM on mental health it has largely been overlooked in DPPs and could impact on uptake and engagement. A recent meta‐analysis indicates that women with GDM are 2–4 times more likely to develop depression during the antenatal or postnatal periods compared to those without GDM (Wilson et al. 2020). Further, a substantial body of qualitative research highlights the emotional and psychological impact of GDM across a range of cultural and healthcare contexts, including distress, fear (Craig et al. 2020; Pham et al. 2022; Van Ryswyk et al. 2015), disordered eating behaviours (Benton, Hotung, et al. 2023), stigma (Davidsen et al. 2022), and the mother‐infant relationship (Benton, Bird, et al. 2023).

Despite a growing body of qualitative research on GDM and DPPs, much of it captures experiences at discrete timepoints, typically during pregnancy or shortly after birth. Few studies follow women's journey's from pregnancy into the postnatal period, limiting our understanding of how women's experiences, needs, motivations, and engagement with healthcare change over time (Neven et al. 2022; Xu et al. 2023; Lau et al. 2023). There is increasing recognition of the need for a life‐course perspective within GDM, for which qualitative longitudinal approaches are particularly well suited, offering nuanced insights into how women navigate GDM across pregnancy and into the postnatal period (Neale 2020).

### 1.1 Aim

This study aims to explore women's psychosocial and healthcare experiences of GDM from pregnancy to 12 months postnatal. Specifically, its longitudinal approach will investigate women's understanding and awareness of DPPs, including when information is currently received, opportunities for enhanced risk communication, and their motivations and barriers for engaging with preventive interventions at different time points. These insights will inform the future co‐design of a culturally sensitive, accessible, and contextually relevant DPP that reflects women's lived experiences across the full perinatal timeline.

## 2 Methods

### 2.1 Design

This is the protocol for an in‐depth qualitative, longitudinal, interview study. This design will allow for a detailed understanding of women's views and experiences of GDM over the perinatal period. The longitudinal approach will enable description of the temporal nature of GDM management and care, its impact on mental and physical health, perinatal experiences including preconception knowledge and behaviours, and health services utilisation over time, whilst enabling rapport to be build between the researcher [MB] and participants. It will allow for the identification of critical moments, exploring not only decision‐making processes related to health behaviours, but also women's preferences regarding what support or information they want and when, thus highlighting optimal points at which DPP components could be effectively delivered and received to maximise uptake.

The research plan has been informed by reviewing relevant literature, our team's previous research in this area, a Patient and Public Involvement and Engagement (PPIE) group, and advice from clinical and methodological experts.

### 2.2 Funding

The study is funded by the National Institute for Health and Care Research [NIHR] through an Advanced Fellowship awarded to Madeleine Benton (ref: NIHR304430). The views expressed in this publication are those of the author(s) and not necessarily those of the NIHR, NHS or the UK Department of Health and Social Care.

### 2.3 Ethical Issues

The research team will draw on their considerable experience in conducting research in GDM and sensitive topics in pregnancy. We have worked closely with the Patient and Public Involvement and Engagement (PPIE) group and clinicians to discuss the acceptability of the research aims and design. Interviews will be organised at a time and place convenient to participants. Consent will be obtained from all participants, and regular checks made that participants are happy to continue. All participants will be provided with information of support agencies throughout.

Research data and patient‐related information will be managed in accordance with relevant regulatory approvals. Ethics approval was granted on 5th November 2024 by King's College London Research Ethics Committee (HR/DP‐24/25‐45503).

### 2.4 Patient and Public Involvement and Engagement

PPIE is integral to the conduct of this study. An advisory group of seven women is involved in this project. The PPIE group has contributed to different aspects of work that have informed this protocol. Four online sessions have been conducted to date (between October 2023 and July 2024) to develop the research question, study material, recruitment plans, interview schedules, and participant retention plan. The PPIE group will continue to be involved throughout the research process.

### 2.5 Theoretical Perspective

#### 2.5.1 Philosophical Underpinning

Understanding the philosophical stance underpinning research is crucial for ensuring clarity and rigour. Ontology, the ‘study of being’, explores the nature of reality, while epistemology examines the nature and scope of knowledge, emphasising how we come to know what we know (Annells 1996; Levers 2013; Taghipour 2014). This study is situated within a post‐positivist research paradigm (Annells 1996; Levers 2013), underpinned philosophically by a critical realist ontology and an objectivist epistemology. Critical realism states that although an objective reality exists independently of human perception, fully understanding this reality requires acknowledgement of how social contexts and conditioning influence our interpretations (Nairn 2012). By adopting critical realism, this research balances recognition of an independent reality with the understanding that our knowledge of this reality is inevitably shaped by social and cultural factors. Epistemological objectivism suggests researchers adhere to principles emphasising objectivity, thus rejecting the notion that reality is purely socially constructed and asserting instead the existence of an objective reality (Greckhamer and Koro‐Ljungberg 2005Greckhamer and Koro‐Ljungberg 2005). This study embraces principles of positionality and critical reflexivity, acknowledging that researchers' personal perspectives, social positions, and inherent biases inevitably influence their interpretations of participants’ narratives, particularly when researchers have not personally experienced the condition being studied, as is the case with the current research team regarding GDM (Berger 2013; Dodgson 2019).

Furthermore, this study draws on principles of qualitative longitudinal research, which foregrounds a processual ontology, one that views time not as a neutral backdrop but as an active dimension of lived experience (Neale 2020). It enables the study of how experiences unfold over time (at multiple timepoints) and through time (by tracing developmental trajectories and turning points), offering insight into both change and continuity (Neale 2020).

The broader scope of this study reflects a core strength of qualitative longitudinal research, which necessitates attention to both individual experience and wider social, structural, and policy contexts. Rather than narrowing inquiry to a fixed phenomenon at a single point in time, this approach situates women's accounts within the evolving realities of the perinatal period and the healthcare systems they navigate. It anchors analysis in participants' lived experiences while attending to the dynamic interplay between micro‐level (personal) and macro‐level (structural) processes. This broader lens is a necessary feature of qualitative longitudinal research, enabling the research to follow participants' evolving experiences and to identify how personal, relational, and systemic factors interact across time (Neale 2020).

#### 2.5.2 Positionality

This work is shaped by the authors' reflections on positionality, acknowledging how their identities and experiences influence the research. Their backgrounds encompass a range of race/ethnicity, gender identities, parental experiences, and expertise in various research methods. The authors come from cross‐disciplinary professional backgrounds with expertise in women's health, psychology, diabetology, anthropology, and psychiatry, who all have a shared interest in improving women's health. The lead researcher conducting interviews (MB) has considerable experience interviewing women in the perinatal period, particularly those who have experienced GDM. The team will endeavour to understand where any preconceived notions and/or biases may be introduced.

### 2.6 Recruitment

Women will be recruited from across the UK through women's health, pregnancy, and GDM communities, as well as charities and community groups that support women from ethnic minority backgrounds and those whose first language may not be English, to ensure geographical spread. A study advertisement, including a link to detailed study information, will be shared directly with these communities and groups. Women interested in participating will first provide their consent and then their contact details, followed by completion of online screening. This questionnaire will collect baseline demographic data (e.g., age, parity, geographic location) required for the sampling framework described below. A member of the research team will then contact potential participants to discuss the study and their involvement. We will apply maximum variation sampling (Palinkas et al. 2015), using criteria to ensure diversity in ethnicity, geographic area, parity, and GDM experiences.

Eligibility criteria include:

- A self‐reported formal diagnosis of gestational diabetes more than 2 weeks prior to the first interview.
- > 16 years of age. No upper age limit.
- Currently live in the United Kingdom.
- Can read and speak in English or a language in which an interpreter is available.

### 2.7 Interview Procedures

Semi‐structured in‐depth individual interviews, recommended by the PPIE group for discussing sensitive topics (e.g. weight and stigma), will occur at three time points. The PPIE group involved in this project identified three critical time points: Timepoint 1 (35 weeks' gestation); Timepoint 2 (3 months postnatal); and Timepoint 3 (12 months postnatal). Each interview is expected to last around 60 min. Previous research has demonstrated the considerable temporal nature of GDM (Benton, Davies, et al. 2023; Benton et al. 2024, 2025), and the PPIE group has suggested that these time points are important in the GDM journey and therefore we believe women will be more likely to engage with the longitudinal nature of this planned qualitative study. Interviewing the same participants across the perinatal period facilitates close observation of how meanings, motivations, and behaviours shift in response to evolving personal, relational, and structural conditions (Neale 2020).

**FIGURE 1:**
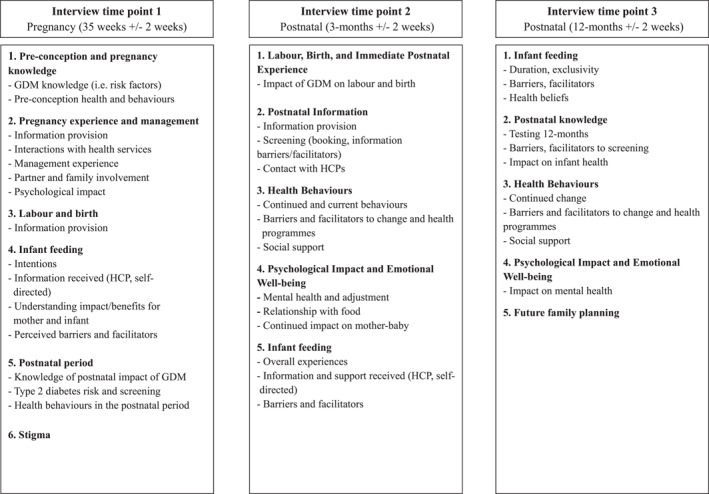
Summary of interview schedule topics.

A flexible approach to conducting interviews will be adopted, allowing women to select the time and location of interviews. Interviews will be conducted face‐to‐face, by telephone, or via video call, reflecting methodological literature indicating that remote interviewing methods can yield data comparable to in‐person interviews and provide greater convenience, particularly for participants caring for newborns (Archibald et al. 2019; Brown 2018; Holt 2010; Lo Iacono et al. 2016). This approach was also informed by recommendations from the PPIE group and practical considerations. Interview schedules (Figure 1) have been developed and piloted, covering thematic areas tailored specifically to each interview time point. The evolving interview schedule, informed by data analysis, will shape follow‐up interviews considering previous exchanges and preliminary themes. Interviews will be carried out by MB, a chartered psychologist with advanced qualitative interviewing skills, and will follow standard protocols for working as a qualitative researcher in the field for sensitive topics (Silverio et al. 2022). All participants will be provided with a £20 gift voucher after each interview to reimburse them for their time. Throughout the study, attention will be paid to building appropriate rapport to support retention without compromising interviewer neutrality. A retention plan, including timely reminders and flexibility in interview scheduling informed by the PPIE group, will also be implemented.

### 2.8 Analysis Plan

All interviews will be recorded and transcribed verbatim, either by the research team or a third‐party transcription service, and anonymised. Field notes will be taken by the interviewer and the research team during the review of interview transcripts, documenting reflections on the interview and any additional interactions, and recorded in a separate Microsoft Word document. NVivo will be used for data handling. Analysis of interview transcripts and field notes will commence alongside data collection, with initial analytical insights informing further data collection. Members of the research team will meet to discuss ongoing analysis and ensure consistency.

Data will be managed and analysed electronically either using Microsoft Word for manual coding or a qualitative data analysis software (e.g., NVivo). We will apply qualitative longitudinal research principles to our analysis by first conducting within‐case analyses, then performing a cross‐case comparison, and finally undertaking an integrative processual analysis to identify key temporal patterns (Neale 2016). Analysis will engage with critical reflexivity throughout, data will be iteratively coded, and accuracy checking will take place when finalising and selecting quotations for inclusion.

## 3 Discussion

### 3.1 Novelty and Strengths of the Proposed Study

The strength of this study lies in the in‐depth, contextual qualitative nature of the data. Our anticipated study population will be diverse in terms of age, parity, ethnic background, location in the UK, GDM experience, and spoken language which will ensure a more representative set of findings can be derived. Furthermore, the range of expertise and experience on the team will ensure we undertake a high‐quality study, with rigorous findings, which can feed directly the development of support and DPPs for women who have experienced GDM.

### 3.2 Dissemination

Findings will be disseminated through peer reviewed journal publications and in presentations at relevant conferences. A lay summary of findings and infographic will be distributed to relevant groups. In addition to traditional academic dissemination, findings will be disseminated via a variety of community‐focused and engagement strategies, closely developed with the PPIE group. Data will inform the co‐development of a DPP for women with GDM. All data will be anonymised before dissemination.

## 4 Conclusion

This protocol outlines the methodological approach for a qualitative longitudinal study investigating women's experiences of GDM from pregnancy until 12 months postnatal. The findings will provide a greater understanding of the psychosocial and healthcare experiences of women with GDM across time. Insights will inform the co‐development of tailored mother‐infant dyad intervention for diabetes prevention.

## Author Contributions

**Madeleine Benton:** conceptualization, funding acquisition, methodology, project administration, supervision, visualization, writing – original draft, writing – review and editing. **Elana Payne:** methodology, project administration, visualization, writing – original draft. **Nabiha Waheed:** writing – review and editing. **Katrina Turner:** conceptualization, writing – review and editing. **Helen R. Murphy:** conceptualization, writing – review and editing. **Helen Skouteris:** conceptualization, writing – review and editing. **Khalida Ismail:** conceptualization, writing – review and editing. **Sergio A. Silverio:** conceptualization, methodology, supervision, writing – review and editing.

## Ethics Statement

Ethical approval was granted on 5th November 2024 by King's College London Research Ethics Committee (HR/DP‐24/25‐45503).

## Consent

The authors have nothing to report.

## Conflicts of Interest

The authors declare no conflicts of interest.

## Permission to Reproduce Material From Other Sources

The authors have nothing to report.

## Data Availability

The data for this study will be made available from the corresponding author upon reasonable request.
